# Emerging Roles of Diacylglycerol-Sensitive TRPC4/5 Channels

**DOI:** 10.3390/cells7110218

**Published:** 2018-11-20

**Authors:** Michael Mederos y Schnitzler, Thomas Gudermann, Ursula Storch

**Affiliations:** 1 Walther Straub Institute of Pharmacology and Toxicology, Ludwig Maximilians University of Munich, 80336 Munich, Germany; thomas.gudermann@lrz.uni-muenchen.de; 2 DZHK (German Centre for Cardiovascular Research), Munich Heart Alliance, 80802 Munich, Germany; 3 Comprehensive Pneumology Center Munich (CPC-M), German Center for Lung Research, 81377 Munich, Germany; 4 Institute for Cardiovascular Prevention (IPEK), Ludwig Maximilians University of Munich, 80336 Munich, Germany

**Keywords:** TRPC channels, diacylglycerol, TRPC4, TRPC5, NHERF

## Abstract

Transient receptor potential classical or canonical 4 (TRPC4) and TRPC5 channels are members of the classical or canonical transient receptor potential (TRPC) channel family of non-selective cation channels. TRPC4 and TRPC5 channels are widely accepted as receptor-operated cation channels that are activated in a phospholipase C-dependent manner, following the G_q/11_ protein-coupled receptor activation. However, their precise activation mechanism has remained largely elusive for a long time, as the TRPC4 and TRPC5 channels were considered as being insensitive to the second messenger diacylglycerol (DAG) in contrast to the other TRPC channels. Recent findings indicate that the C-terminal interactions with the scaffolding proteins Na^+^/H^+^ exchanger regulatory factor 1 and 2 (NHERF1 and NHERF2) dynamically regulate the DAG sensitivity of the TRPC4 and TRPC5 channels. Interestingly, the C-terminal NHERF binding suppresses, while the dissociation of NHERF enables, the DAG sensitivity of the TRPC4 and TRPC5 channels. This leads to the assumption that all of the TRPC channels are DAG sensitive. The identification of the regulatory function of the NHERF proteins in the TRPC4/5-NHERF protein complex offers a new starting point to get deeper insights into the molecular basis of TRPC channel activation. Future studies will have to unravel the physiological and pathophysiological functions of this multi-protein channel complex.

## 1. Introduction

Transient receptor potential classical or canonical 4 (TRPC4) and TRPC5 channels belong to the transient receptor potential classical or canonical (TRPC) cation channel subfamily, which comprises seven members. TRPC channels are regarded as non-selective, receptor-operated cation channels that are important for calcium homeostasis. They are activated via the G_q/11_-signaling cascade as a function of phospholipase C (PLC) [1]. Moreover, the TRPC4 and TRPC5 channels can also be activated following the G_i/o_-protein coupled receptor activation [2,3,4]. However, the mechanism of the G_i/o_-mediated TRPC4/5 channel activation is still not completely understood and might involve either a direct G_i/o_-protein interaction [3], or the activation of the PLC isoform, PLCδ1 [2]. Even a G_s_-protein mediated activation mechanism was proposed for the TRPC5 channels [5]. Therefore, elevating the intracellular cyclic adenosine monophosphate (cAMP) levels might potentiate the TRPC5 currents by increasing the channel trafficking to the plasma membrane. Additionally, a store-operated activation mechanism for the TRPC4 [6,7,8,9] and TRPC5 channels [10,11,12] was proposed, and is still under discussion [13,14]. This article will only focus on the G_q/11_-protein mediated signaling pathway leading to the TRPC4 and TRPC5 channel activation.

All of the TRPC channels are tetramers formed by four TRPC protein subunits, which was confirmed recently by structural analysis [15,16,17,18]. TRPC proteins can form not only homotetrameric but also heterotetrameric cation channels [19,20,21,22] with distinct channel properties, such as altered calcium permeability [21,23,24,25]. Because of their sequence homology, the TRPC channel family can be divided into the following subgroups: TRPC1, TRPC4/5, and TRPC3/6/7. TRPC2 has a special role as it represents a pseudogene in humans that is not functionally expressed because of several stop codons in the open reading frame [26,27]. However, in rodents, the TRPC2 channels are functionally expressed (e.g., in the olfactory cells of the vomeronasal organ, where they are important for pheromone sensing [28]).

Although the G_q/11_-protein mediated TRPC channel activation is widely accepted, the precise signaling pathway resulting in the channel opening has remained elusive for quite some time. The activation of the G_q/11_-protein coupled receptors leads to the activation of PLC, which cleaves phosphatidylinositol-4,5-bisphosphate (PIP_2_) into the second messengers, inositol-1,4,5-trisphopshate (IP_3_) and 1,2-diacyl-*sn*-glycerol (DAG), and to an oxonium ion [29,30]. There is broad agreement that the TRPC3/6/7 subfamily is directly activated by DAG, the cleavage product of PIP_2_ [31]. However, IP_3_ might also contribute to the activation of endogenously expressed TRPC7 channels [32]. Notably, a DAG binding site has not been identified until now, and it is not clear whether DAG directly activates the channel or whether it first interacts with an additional protein, which in turn causes the channel activation. Unfortunately, the recent structural analysis of the TRPC3, TRPC4, and TRPC6 channels only displays a closed channel conformation [15,16,17,18]. However, the structural model of TRPC3 reveals two lipid-binding sites, one being sandwiched between the pre-S1 elbow and the S4–S5 linker, and the other being close to the pore-forming domain, where the conserved “LWF” motif of the TRPC family is located [16]. Perhaps these lipid binding sites reflect potential DAG binding sites. Interestingly, it was recently reported that the exchange of a highly conserved amino acid located close to the pore-forming domain, affects the DAG recognition of the TRPC3 channels [33]. Thus, the second lipid binding site might indeed reflect a potential DAG binding site. TRPC2 channels are also regarded as being DAG sensitive [34,35]. In contrast to the TRPC2, TRPC3, TRPC6, and TRPC7 channels, the TRPC4 and TRPC5 channels were commonly considered as DAG insensitive [31], as DAG or the membrane permeable DAG analogue 1-oleoyl-2-acetyl-sn-glycerol (OAG) even inhibited the basal TRPC5 currents [36]. Interestingly, Venkatachalam and colleagues showed that the DAG-induced TRPC5 channel inhibition is related to the protein kinase C (PKC) activation [36]. Moreover, it was demonstrated that the homotetrameric TRPC4 and TRPC5 channels are activated by PIP_2_ depletion [37,38]. In contrast, the heterotetrameric TRPC1/4 and TRPC1/5 [39] and homotetrameric TRPC6 and TRPC7 channels [40] are instead inhibited by the PIP_2_ depletion. These contradictory findings suggest that the TRPC channel-lipid interaction is rather complex. Another unique structural feature of the TRPC4 and TRPC5 channels is their capability to interact with the PDZ I domain of the scaffolding proteins Na^+^/H^+^ exchanger regulatory factor 1 and 2 (NHERF1 and NHERF2) [41,42,43]. The NHERF1 and NHERF2 proteins are structurally related; can form homodimers [44]; and possess two PDZ binding domains as well as a C-terminal binding domain, which enables crosslinking with the actin cytoskeleton via ezrin, radixin, and moesin proteins [41,45]. Thus, NHERF1 and NHERF2 proteins are commonly regarded as adapter proteins that crosslink integral membrane proteins with the cytoskeleton, thereby increasing their membrane localization [46,47,48].

## 2. DAG-Mediated Activation Mechanism of TRPC4 and TRPC5 Channels

The first evidence that the TRPC5 channels might be DAG sensitive was presented by Lee and colleagues, who performed electrophysiological whole-cell measurements on murine gastric smooth muscle cells endogenously expressing TRPC5 channels, and found that the channels are activated by OAG [49]. However, the mechanistic insights into the DAG mediated TRPC5 channel activation were missing. A remarkable structural difference between TRPC4 and TRPC5, and the well characterized DAG sensitive TRPC3/6/7 channels, is the PDZ binding motif with the amino acid sequence “VTTRL” at the very end of the C-terminus [41,42,50]. This PDZ binding motif includes a potential PKC phosphorylation site, which was identified as being important for the TRPC5 current inactivation following the receptor activation [51]. The amino acid exchange from threonine to alanine at position 972 (T972A) in the murine TRPC5 channels resulted in a loss of current desensitization during the receptor activation with carbachol [51]. Thus, this was the first evidence that the PKC phosphorylation might regulate the TRPC5 channel function [36,51].

The C-terminal PDZ binding motif allows for interactions with the adapter proteins NHERF1 and NHERF2. This C-terminal TRPC4/5 interaction was demonstrated several times by performing co-immunoprecipitations and functional studies using the patch-clamp technique [41,42,43]. However, only a slight enhancement of the membrane expression was found to be similar to what was observed when analyzing the chloride channel cystic fibrosis transmembrane conductance regulator (CFTR) [52] and other integral membrane proteins. Thus, the functional implications of the TRPC4/5-NHERF interaction were missing. Interestingly, our recent findings indicate that the TRPC4 and TRPC5 channels are DAG-sensitive similar to other TRPC channels [53]. However, in contrast to TRPC3/6/7, their DAG-sensitivity is tightly regulated by the C-terminal NHERF1 and NHERF2 interaction. The G_q/11_-protein coupled receptor activation causes a cleavage of PIP_2_, resulting in a conformational change of the C-terminus, which in turn causes the dissociation of the NHERF proteins from the C-terminus of the channel. This dynamic dissociation was monitored employing the method of dynamic intermolecular fluorescence resonance energy transfer (FRET) between fluorescence tagged TRPC5 and NHERF proteins. The separation of the NHERF proteins from the C-terminus was a prerequisite for DAG sensitivity. Moreover, the C-terminal NHERF interaction strongly depended on the PKC phosphorylation status of the C-terminal PDZ binding motif “VTTRL” [53]. The PKC inhibition resulted in the DAG sensitivity, and the PKC phosphorylation mutant T972A of murine TRPC5 was sensitive to DAG, suggesting that PKC phosphorylation is a prerequisite for NHERF binding, thereby suppressing the DAG sensitivity. Thus, the NHERF proteins are dynamic regulators of the TRPC4 and TRPC5 channel activity. This signaling pathway was also observed in the primary cell lines (e.g., in proximal tubule cells and in hippocampal neurons, which endogenously express TRPC4 and TRPC5 channels, respectively) [53]. This activation model has the potential to integrate the contradictory findings of different research groups concerning the TRPC4 and TRPC5 channel activation. Therefore, the pieces of the puzzle like the PLC dependent receptor-mediated TRPC4/5 channel activation [1], the inhibitory effect of DAG or DAG analogues [31,36] on the native TRPC4/5-NHERF-channel complex [41,42,43], the activation by PIP_2_ depletion [37,38], the inhibitory effect of PKC phosphorylation on DAG sensitivity [36], and the DAG sensitivity of the endogenous gastric TRPC5 channels [49] coalesce into a consistent picture. These findings lead to a new concept, that all of the TRPC channels are DAG-sensitive, and that classifying the TRPC channels as DAG-sensitive and -insensitive channels should be avoided. Consequently, a common DAG binding site for TRPC channels can be proposed. Perhaps the highly conserved amino acid near the TRPC pore domain that affects the DAG activation [33] participates in DAG binding.

**Figure 1:**
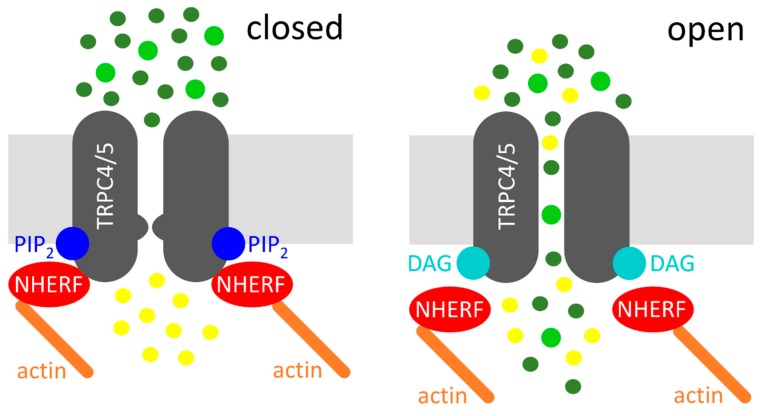
Diacylglycerol (DAG)-mediated activation of transient receptor potential classical or canonical 4/5 (TRPC4/5) channels. Left: Na^+^/H^+^ exchanger regulatory factor (NHERF) proteins and phosphatidylinositol-4,5-bisphosphate (PIP_2_) interact with the C-termini of TRPC4/5, which depicts the closed state of the channel. Right: receptor activation (not displayed) leads to the cleavage of PIP_2_, resulting in the dissociation of NHERF and in DAG binding, which represents the open state of the channel. Sodium cations (dark green circles), calcium cations (light green circles), and potassium cations (yellow circles) are displayed. The potassium efflux is mainly relevant in the excitable cells.

Altogether, a new model of G_q/11_-protein mediated TRPC4 and TRPC5 channel activation can be proposed. The agonist-induced G_q/11_-protein coupled receptor activation causes the activation of PLC, which in turn cleaves PIP_2_ into the two second messengers IP_3_ and DAG. The PIP_2_ cleavage causes a conformational change at the C-terminus of TRPC4 and TRPC5, which results in a dissociation of the NHERF proteins from the C-terminus, thereby evoking a DAG-sensitive channel conformation. Then, the PIP_2_ cleavage product DAG can activate the channel. This model is illustrated in Figure 1.

## 3. Physiological and Pathophysiological Roles of NHERF Proteins

NHERF1 and NHERF2 proteins belong to a family of scaffolding proteins that crosslink the integral membrane proteins with the cytoskeleton. It is commonly accepted that NHERF proteins increase the membrane localization of several membrane proteins, like transporters, receptors, and ion channels [46,47,48]. However, besides their anchoring function, the NHERF proteins are of the utmost importance for the maintenance of essential cellular functions (e.g., in the kidney or in the small intestine, where they interact with transporters, ion channels, signaling proteins, transcription factors, enzymes, G-protein coupled receptors, and tyrosine kinase receptors) [47,48,54,55,56]. Thus, NHERF proteins are involved in numerous physiological processes. For example, in proximal tubule cells, NHERF proteins regulate phosphate transport [57]. A mutation in the PDZ I domain of human NHERF1 was found in patients with impaired renal phosphate reabsorption due to a reduced expression of the renal phosphate transporter NPT2a [58]. In astrocytes, NHERF proteins regulate the activity of the glutamate transporter (GLAST) and of the metabotropic glutamate receptor (mGlu5) [59,60], and in the small intestine, they control ion transport via interactions with the Na^+^/H^+^ exchanger (NHE3) [61]. Moreover, the mice lacking NHERF1 and adult humans harboring NHERF1 mutations suffer from osteopenia [62,63], which might be due to abnormal osteoblast differentiation [64]. Furthermore, NHERF proteins influence proliferation [65,66], and they may be involved in carcinogenesis and in the progression of liver, breast, and colon cancer; small-cell lung carcinoma; and glioblastoma [67,68,69,70]. A mutation in the PDZ I domain of NHERF1 was identified in the patients with medullar breast carcinoma [71]. This mutation resulted in a reduced interaction of NHERF1 with the epidermal growth factor receptor, thereby promoting the progression of breast cancer. Another mutation in the PDZ II domain of human NHERF1 resulted in a nuclear translocation of NHERF1, thereby inducing carcinogenesis [72]. Recently, in the tumors from ovarian cancer patients, a mutation in the PDZ II domain of NHERF1 was identified, which might contribute to the disease progression [73]. These data suggest that wild-type NHERF1 may act as a tumor suppressor. The subcellular NHERF expression also plays a critical role in cancer cells. In breast cancer cells, the subcellular NHERF expression might even serve as a prognostic marker, as a high cytoplasmic expression of NHERF was associated with a high aggressiveness and poor prognosis [74]. Furthermore, aberrant nuclear NHERF1 expression might be important for the carcinogenesis and progression of colon [74] and breast cancer [72].

The specific role of NHERF proteins for channel function is poorly understood. Beside the regulatory role of NHERF proteins on TRPC4 and TRPC5 channel function [53], the interaction with NHERF was identified as playing an important role in the proper function of the CFTR chloride channel [52]. Mutations in the CFTR channel are known to cause cystic fibrosis [75], which is characterized by the accumulation of viscous mucus, because of impaired fluid transport. Furthermore, CFTR mutations can cause congenital absence of the vas deferens and male infertility [76]. Interestingly, the NHERF2 interaction with the lysophosphatidic acid receptor 2 (LPAR2) promotes the assembly of CFTR–NHERF2–LPAR2 protein complexes, which results in an impaired CFTR function [77,78]. The disruption of this protein complex might enhance the CFTR function of patients suffering from cystic fibrosis [52]. Altogether, the NHERF proteins or their protein complexes might be interesting novel targets for the treatment of diseases

## 4. Physiological and Pathophysiological Roles of TRPC4 and TRPC5 Channels

TRPC4 and TRPC5 channels are expressed in several cells and tissues (e.g., in the brain [79,80,81,82,83], kidney [80,84,85], and vascular system [7,8,86]). In particular, the TRPC4 and TRPC5 channel expression is very high in the central nervous system [7,79,82]. Here, the TRPC4 and TRPC5 channels are involved in the amygdala function and account for fear-related behavior against aversive stimuli [87,88]. In addition, the TRPC4 and TRPC5 channels are important for peripherally induced neuropathic pain behavior. Microinjections of the TRPC4 and TRPC5 channel blocker ML-204 into the amygdala of rats reduced the sensory and the affective pain sensitivity [89]. Thus, in the future, TRPC4/5 blockers that are able to cross the blood–brain barrier might be used as novel anxiolytics or even as innovative analgesics against peripheral neuropathies. Furthermore, the TRPC4 and TRPC5 channels are expressed in the dorsal root ganglia, where they contribute to axonal regeneration after nerve injury [90], to itching [91], and to cold detection [92].

Moreover, in the hippocampal CA1 pyramidal cells from rats, the calcium and sodium influx through the TRPC5 channels generated a plateau potential [93] that is also observed during epileptic seizures [94,95]. In accordance with this neurophysiological evidence, the TRPC5 gene-deficient mice exhibited significantly reduced seizures. Thus, future studies will have to show whether TRPC5 channels represent interesting novel target structures for the treatment of epileptic disorders.

Furthermore, the TRPC5 channels reduce the hippocampal neurite length and growth cone morphology by reducing the filopodia length growth, which leads to impaired axon guidance [96]. The TRPC5 channels also play a role in podocytes and in fibroblasts. Interestingly, in these cells, the TRPC5 and TRPC6 channels have opposite effects on the actin cytoskeleton [97]. The receptor-operated TRPC5 channel activation by angiotensin II decreased the number of parallel stress fibers via the activation of the small guanosine-5′-triphosphate (GTP)ase protein Rac1, leading to a motile and non-contractile phenotype in vitro, while the TRPC6 activation by angiotensin II increased the formation of parallel stress fibers via the activation of the small GTPase protein Rho A, resulting in a contractile but non-motile phenotype [97]. These differential channel functions might be due to a specific subcellular localization of TRPC5 and TRPC6 channels in podocytes, or be caused by a distinct signaling elicited by the podocyte-specific TRPC6 and slit membrane protein channel complex [98,99]. The reorganization of the actin cytoskeleton characterized by a reduction of the parallel stress fibers results in podocyte injury, leading to the loss of podocyte foot processes, which in the end results in the disruption of the slit diaphragm and in massive proteinuria [100,101,102]. Thus, the TRPC5 channel blockers might be useful for the treatment of podocyte diseases by preventing end-stage renal disease [103,104].

In addition, the TRPC4 and TRPC5 channels might play a pathophysiological role in cancer cells. The increased TRPC5 channel activity in breast cancer [105] and in colorectal cancer cells [106] caused an increased expression of the ABC transporter P-glycoprotein (MDR1). MDR1 is an important molecular correlate for drug-resistance against chemotherapeutic agents. For example, MDR1 eliminates the well-known and commonly used DNA intercalating drug doxorubicin, the tubulin-targeting drug paclitaxel, and the antimetabolite 5-fluorouracil. In breast cancer cells, the transcription factor NFATc3, and in colorectal cancer cells, the structural protein and transcription factor β-catenin, are thought to enhance the MDR1 expression [105,106]. In contrast, the potent TRPC4 and TRPC5 channel activator (-)-englerin A showed pronounced cytotoxic effects on diverse cancer cell lines, with an EC_50_ value of ~20 nM [107,108]. (-)-englerin A was even effective on triple-negative breast cancer cells [109], which do not express the drug targets of estrogen, progesterone, and human epithelial growth factor (HER2) receptor. Of all breast cancer patients, 15% have triple-negative breast cancer [110], which is regarded as very aggressive [111]. The main treatment is surgery, but specific targets for target-orientated chemotherapeutic agents are missing, and patients suffer from frequent relapses within the first three years [112]. A more targeted medical treatment would be of great benefit for these patients. Notably, although (-)-englerin A was selectively cytotoxic to cancer cell lines, adverse reactions were observed in mice and rats after (-)-englerin A injections [108], which were mediated by the TRPC4/5 channels [113]. Thus, the therapeutic application of (-)-englerin A might be limited.

There is evidence that the TRPC5 channel activity increases angiogenesis in cases of breast cancer by the activation of the transcription factor hypoxia-inducible factor 1 (HIF-1), leading to vascular endothelial growth factor (VEGF) formation [114], thereby promoting cancer growth. In contrast, it was reported that the TRPC4 channel activation reduces angiogenesis in cases of renal cell carcinoma cells by the secretion of the inhibitor of angiogenesis thrombospondin-1 [115]. Moreover, the TRPC4 and TRPC5 channel activation in the endothelial cells increases vasculogenesis [116,117], indicative of a pro-angiogenetic effect of these channels. Furthermore, the increased expression of TRPC1, TRPC3, TRPC4, and TRPC6 in ovarian cancer cells increased the migration and proliferation, and therefore had a tumorigenic effect [118]. Thus, other TRPC channels, like TRPC1, TRPC3, and TRPC6, might also function as targets for chemotherapeutic agents. However, at present, the majority of publications point to the TRPC4 and TRPC5 channels as potential new drug targets [119].

The novel role of the NHERF adapter proteins as dynamic regulators of the TRPC4 and TRPC5 channel activity [53,120] might also be important for several other physiological or pathophysiological processes. Interestingly, the NHERF1 protein/channel interaction is of the utmost importance for the proper function of CFTR channels. The NHERF1 proteins stabilized and enhanced the membrane expression of the misfolded CFTR mutant channels [121], which partially restored the channel activity after the NHERF binding. 

The TRPC4 and TRPC5 channels, and the NHERF proteins are co-expressed in various excitable and non-excitable cells and tissues. Hence, it can be speculated that the inhibitory effect of the NHERF interaction on the TRPC4/5 channel function may contribute to several physiological or pathophysiological conditions. For example, in tumor cells, the TRPC4/5 channels and the NHERF proteins and NHERF mutations account for cancer progression. However, the effect of the TRPC4/5-NHERF protein complex on tumor growth has largely remained elusive until now. The NHERF interaction also inhibited the DAG mediated TRPC4/5 channel activation in murine hippocampal neurons and in proximal tubule cells [53], suggesting a regulatory role. Altogether, further studies will be needed to show whether the TRPC4/5-NHERF protein complexes have the potential to serve as novel target structures for therapeutics.

## 5. Conclusions

The new concept that all TRPC channels are DAG sensitive is not only a new starting point for deeper insights into the activation mechanism of TRPC channels on a molecular level, but it might also help to unravel the physiological and pathophysiological roles of these channels, which have not been fully understood until now. On the molecular level, the conformational changes and the kinetics of the conformational changes leading to the TRPC4 and TRPC5 channel activation are largely elusive, as a structure analysis only revealed the inactive channel states. Moreover, the role of the NHERF proteins as dynamic regulators of the TRPC4 and TRC5 channel activation sheds new light on the function of ion channels and adapter proteins in multi-protein complexes. In the TRPC4/5-NHERF protein complexes, the NHERF proteins suppress the DAG sensitivity. Thus, for screening purposes, wildtype TRPC5 channels as well as DAG sensitive PKC phosphorylation site mutant T972A, which cannot interact with NHERF, might be used. Without the inhibitory NHERF binding, it can be speculated that other hits will be identified. As the expression pattern of NHERF proteins and TRPC4/5 channels is altered in several cancer cell lines and might be linked to cancer progression, the TRPC4/5–NHERF channel complexes should also be reconsidered as potential novel targets for cancer therapeutics. Future studies will have to unravel the physiological and pathophysiological roles of these channel complexes. However, a thorough analysis of the TRPC channel functions on a molecular level is of the utmost importance to lay the foundation for a better understanding of the role of TRPC channels in health and disease.
